# COHORT DYNAMICS OF SOCIAL SUPPORT EXCHANGE: INSIGHTS FROM DIVERSE AGING IN THE US

**DOI:** 10.1093/geroni/igae098.0603

**Published:** 2024-12-31

**Authors:** Bianca Suanet, Oliver Huxhold, Jacqui Smith

**Affiliations:** Vrije Universiteit Amsterdam, Amsterdam, Noord-Holland, Netherlands; German Centre of Gerontology, Berlin, Berlin, Germany; University of Michigan, Ann Arbor, Michigan, United States

## Abstract

**Background:**

Studies from Europe indicate an increase in social support exchanges among older adults, but whether this trend also applies to the US and the perception of these exchanges as positive or negative remains unclear. Furthermore, it’s uncertain if these changes also benefit those that face disadvantage, such as low income and racial minority groups, especially when considering intersecting factors.

**Methods:**

This study employs cohort-sequential analysis of Health and Retirement Study (HRS) data collected from 2008 to 2022 in 8 waves to investigate cohort differences in positive and negative social support exchanges among older adults in the United States. It focuses on the influences of cohort, race, and income on social support dynamics, employing data from the psycho-social questionnaire.

**Results:**

Despite a general increase in positive support exchanges, disparities in social support exchange persist based on race and socioeconomic status. Individuals with lower incomes and from ethnic minority backgrounds experience disproportionately more negative social support exchanges, indicating persistent inequalities.

**Discussion:**

Recognizing the complexities of social support exchanges, particularly in relation to cohort, race, and socioeconomic status, is crucial for policymakers and healthcare professionals. Tailored interventions are essential to address disparities and promote equitable aging outcomes and overall well-being among older adults in the US.

